# Comparison of Cantharidin Toxicity in Breast Cancer Cells to Two Common Chemotherapeutics

**DOI:** 10.1155/2014/423059

**Published:** 2014-09-14

**Authors:** Katie M. Kern, Jennifer R. Schroeder

**Affiliations:** Department of Biology, Millikin University, Decatur, IL 62522, USA

## Abstract

As part of a larger study synthesizing a more directed form of chemotherapy, we have begun to assess the efficacy of different potential toxins that could be delivered locally rather than systemically. In doing so, we hope to reduce the systemic side effects commonly observed, while maintaining a high level of toxicity and eliminating the need for metabolic alterations. In a search for this more efficient method for killing cancerous cells, we have begun studying cantharidin, a toxin used in traditional Chinese medicine, as a potential chemotherapeutic. Using an MTT cell viability assay, the toxicity of cantharidin was compared to both cyclophosphamide and paclitaxel in three different breast cancer cell lines: MCF-7, MDA-MB-231, and SK-BR-3. Increasing the concentration of chemotherapy drugs did decrease cell viability in all cell lines when cantharidin and cyclophosphamide were applied; however differences for paclitaxel were cell-specific. Additionally, cantharidin exhibited the highest decrease in cell viability regardless of cell type, indicating it may be a much more potent and less specific chemotherapeutic. These results will help us move forward in developing a potentially more potent treatment for breast cancer that might eliminate the need for subtype-specific treatments.

## 1. Introduction

Breast cancer is the second leading cause of cancer related deaths in women [1]. While several targeted chemotherapeutics have been developed, the use of these drugs is limited to those patients who exhibit the proper cellular profile, such as triple negative or HER2 positive [2–7]. In lieu of these targeted therapies, a more nonspecific treatment must be utilized. Cyclophosphamide and paclitaxel are two commonly used chemotherapeutics for breast cancer [8]. Cyclophosphamide is an alkylating agent from a class of nitrogen mustards that will damage DNA, thus preventing cancer cells from reproducing [9, 10]. Paclitaxel interferes with the cell cytoskeleton by altering microtubule rearrangement [11]. Paclitaxel was the first taxane used in clinical trials and found to be used with ovarian and breast cancers resistant to chemotherapy and has been used* in vivo* studies for inhibiting mammary cancer growth, delaying mammary adenocarcinomas, and decreasing tumor angiogenesis [12]. Usage of paclitaxel with radiation, in initial therapy, was shown to improve survival [13].

Overall, both of those toxins have produced good outcomes; however when these toxins are used they are injected intravenously and spread throughout the whole body, indiscriminately causing death to both cancerous and noncancerous cells [14–17]. Commonly reported side-effects include hair loss, bladder damage, joint pain, and anemia [18, 19]. Additionally, while these drugs have typically been used for nonspecific treatment of breast cancer, they may in fact show mixed efficacy depending on the genetic background of the tumors, with some tumors now showing paclitaxel resistance [20–22]. It is our long-term goal to identify compounds which might exhibit higher levels of cell death while having reduced specificity and develop a mechanism for a more localized delivery of these compounds to reduce systemic toxicity.

Therefore, we have decided to examine cantharidin, a natural toxin that can induce apoptosis. Cantharidin is terpenoid that is secreted by several species of beetles of the Meloidae family, primarily the blister beetles, cardinal beetles, and soldier beetles, along with the Spanish fly* Lytta vesicatoria* [23]. It has been used extensively in traditional Chinese medicine as a topical treatment for MCV infections and warts [24]. Recent studies have indicated that cantharidin may act via inhibition of protein phosphatases I and IIA, activation of p53, inhibition of CDK1, and production of reactive oxygen species [24–27]. However, as cantharidin is a potent inducer of apoptosis, its safety as an anticancer agent is questionable. Li et al. [28] reported cantharidin selectivity in inducing cell death in pancreatic cancer over normal pancreatic cell lines, and cantharidin has shown potential for use in the treatment of colon and liver cancers [29, 30]; it is also highly toxic to the gastrointestinal tract and kidneys which can result in mortality [31, 32].

Our long-term goal is to create a chemotherapeutic that could be utilized locally to kill cancerous cells while leaving the surrounding tissues and rapidly dividing cells such as hair follicles and red blood cells intact. As part of this goal, we seek to identify a compound that exhibits a more rapid and complete toxicity than the currently available choices. It is our hypothesis that one of the toxins we are studying will clearly be more toxic than the other two and thus will be an ideal choice for moving forward in the development of new chemotherapy treatments. To compare the cytotoxicity of these potential chemotherapies, and since different cell backgrounds may result in different responses to toxin exposure, we have used three cancer cell models: MCF-7 cells, MDA-MB-231 cells, and SK-BR-3 cells. We applied cantharidin, cyclophosphamide, or paclitaxel to these cells for up to 96 hours and assessed cell viability. The results of these studies are presented herein.

## 2. Materials and Methods

### 2.1. Cell Culture

Three mammary adenocarcinoma cell lines were utilized: SK-BR-3 cells, which overexpress the Her2 gene, MDA-MB-231 cells, which are estrogen-receptor negative, and MCF-7 cells, which express the estrogen receptor. SK-BR-3 and MDA-MB-231 cells were maintained in DMEM/F12 media (Life Technologies, Grand Island, NY) supplemented with antibiotics (50 IU/mL penicillin, 50 *µ*g/mL streptomycin, and 5 *µ*g/mL gentamycin sulfate) and 10% newborn calf serum (Life Technologies, Grand Island, NY). MCF-7 cells were maintained in Eagle's modified essential medium (HyClone, GE Healthcare Bio-Sciences, Pittsburgh, PA) containing antibiotics as above and 5% calf serum (PAA, GE Healthcare Bio-Science Corp., Piscataway, NJ). All cells were maintained at 37°C in a 5% CO_2_ humidified environment.

### 2.2. MTT Assay

When cells reached a minimum of 75% confluency, they were washed with HBSS, trypsinized, resuspended in media, and plated into one 96-well plate per original 75 cm^3^ flask. After twenty-four hours, a set of plated cells was treated in triplicate in the respective media containing either 1 to 100 *μ*M cantharidin, 0.1 to 10 *µ*M paclitaxel, or DMSO vehicle control, or 10 to 1000 *μ*M cyclophosphamide or H_2_O vehicle control. This treatment was repeated on fresh cells after 24, 48, and 72 hours.

After a maximum of 96 hours of treatment, an MTT (3-(4,5-dimethylthiazol-2-yl)-2,5-diphenyltetrazolium bromide, Sigma, St. Louis, MO) based cell viability assay was performed in the manner of Sargent and Taylor [33]. Briefly, MTT was added to each well to a final concentration of 0.5 mM, and cells were incubated at 37°C in a 5% CO_2_ incubator. After 3 hours, the MTT solution was removed and cells were solubilized in 200 *μ*L of DMSO. The amount of stain was quantified using a microplate reader at 570 nM. The data recorded represented the amount of viable cells in a well and were normalized to the untreated control wells for each experiment. A minimum of four experimental replicates were performed. Univariate analysis of variance (ANOVA) was performed using SPSS (IBM SPSS Statistics 21, IBM Corp., Armonk, NY).

## 3. Results and Discussion

MDA-MB-231, SK-BR-3, and MCF-7 breast cancer cells were exposed to a single chemotherapeutic drug at a range of concentrations spanning the IC_50_ for up to 96 hours. Viable cells were stained with MTT dye, which was converted to formazan by mitochondria in live cells [33]. The amount of formazan was then quantified using a microplate reader. Results were compared to the appropriate control, either DMSO or H_2_O. Statistical analysis of the resulting data was performed using SPSS. Univariate ANOVA indicated cross-interactions between all multifactorial combinations of cell type, toxin, concentration, and time of exposure, and thus smaller interactions (toxin, concentration, and time of exposure plus multifactorial combinations) were examined within each cell type when appropriate.

**Figure 1:**
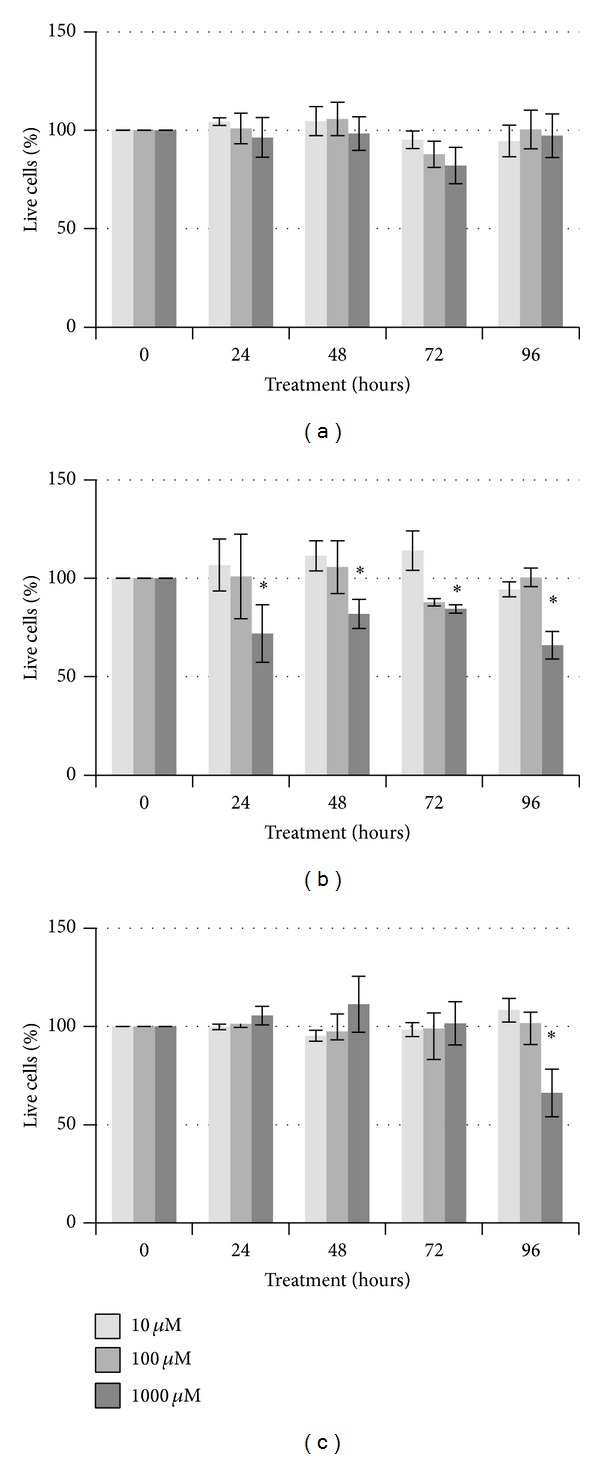
Viability of breast cancer cells after exposure to 10 *µ*M, 100 *µ*M, or 1000 *µ*M cyclophosphamide. MDA-MB-231 (a), SK-BR-3 (b), or MCF-7 (C) cells were treated for up to 96 hours with either cyclophosphamide or a water control. Significant differences compared to the vehicle control (*P < 0.05) are indicated.

Cyclophosphamide is commonly utilized for the treatment of several cancers and is most potent after conversion to its active form by the liver [34]. We selected a treatment range of 10 to 1000 *µ*M or a water vehicle control, as these levels overlap or exceed the reported IC_50_ range of 0.2–17 *µ*M [35, 36]. Cells that were treated with cyclophosphamide showed minimal cell death (Figure 1). No significant differences were observed with up to 1000 *µ*M cyclophosphamide in the MDA-MB-231 cells (Figure 1(a)). In contrast, at this highest concentration, both the SK-BR-3 and the MCF-7 cells showed a reduction in cell viability when compared to the water control. In the SK-BR-3 cells, only 30% death was observed consistently at the highest concentration (Figure 1(b)). A similar difference was only observed at the longest treatment in the MCF-7 cells (Figure 1(c)). Thus, the MCF-7 and MDA-MB-231 cells were more resistant to cyclophosphamide than the SK-BR-3 cells. This was somewhat expected with our direct application of the toxin, as cyclophosphamide is not as toxic without being metabolized by the liver [37]. Franke et al. [38] showed that apoptosis nearly doubled in MCF-7 cells treated with between 0.001 and 1 *µ*M compared to control-treated cells, yet little effect was observed in MDA-MB-231 cells. Even with much higher concentrations of cyclophosphamide, we failed to detect similar levels of cell death.

**Figure 2:**
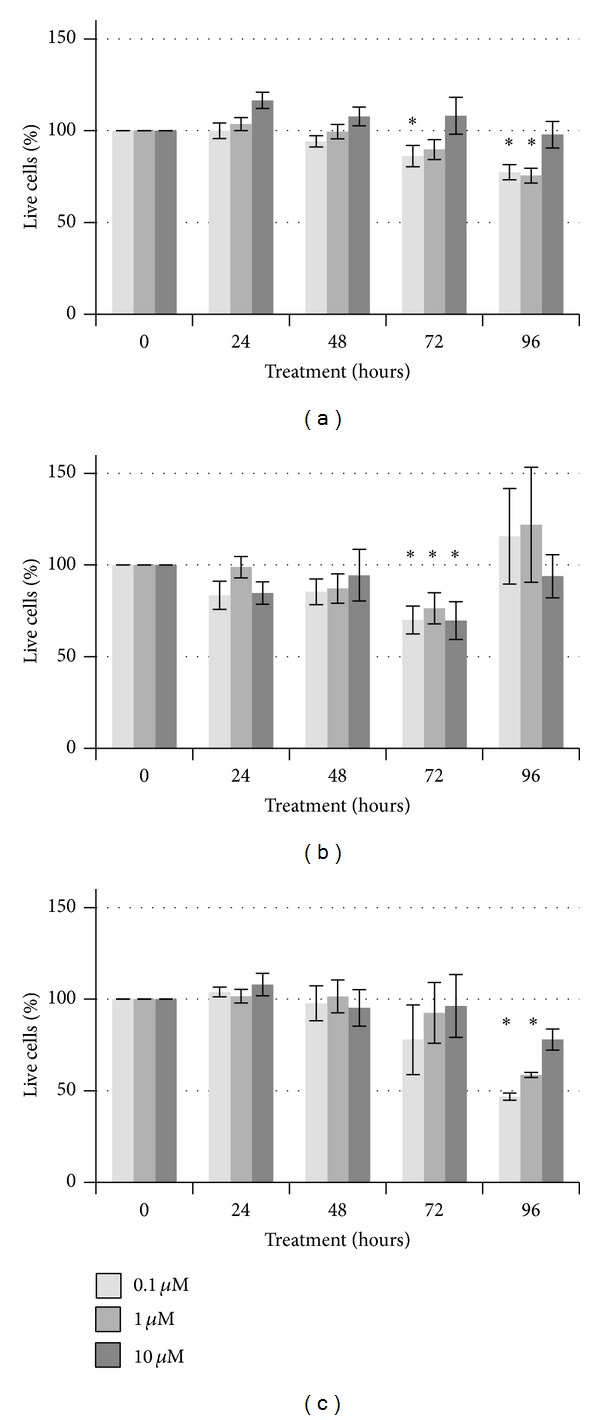
Viability of breast cancer cells after exposure to 0.1 *µ*M, 1 *µ*M, or 10 *µ*M paclitaxel. MDA-MB-231 (a), SK-BR-3 (b), or MCF-7 (c) cells were treated for up to 96 hours with either paclitaxel or DMSO vehicle control. Significant differences compared to the vehicle control (*P < 0.05) are indicated.

Paclitaxel (Taxol) is another chemotherapeutic which is widely used in breast cancer treatments [39–41]. Paclitaxel has shown mixed efficacy dependent upon cell type as well, with an IC_50_ range of 0.02–100 nM [42–45]. Thus, we felt that it was more appropriate to aim for the higher end of this range to ensure cell death in our three cell lines. However, even at this higher dosage, paclitaxel showed limited toxicity (Figure 2). No significant differences in viability were observed at the 24- or 48-hour time points in any of the three cell lines, regardless of concentration used. In MDA-MB-231 cells, we did observe minor reductions in viability at 72 and 96 hours, although this was seen around the middle concentration of 1 *µ*M (Figure 2(a)). In the SK-BR-3 cells, recovery was observed after a temporary decrease in viability at 72 hours (Figure 2(b)). In the MCF-7 cells, a significant effect of time was observed as the 96-hour treatment showed lower survival than the vehicle control for both of the lower concentrations. Recent work has indicated that some cell types may be resistant to paclitaxel due to downregulation of LZTS1 [45, 46]. With the minute toxicological response we detected, combined with the resistance to paclitaxel now being observed, paclitaxel does not appear to be a good selection for a local chemotherapeutic.

**Figure 3:**
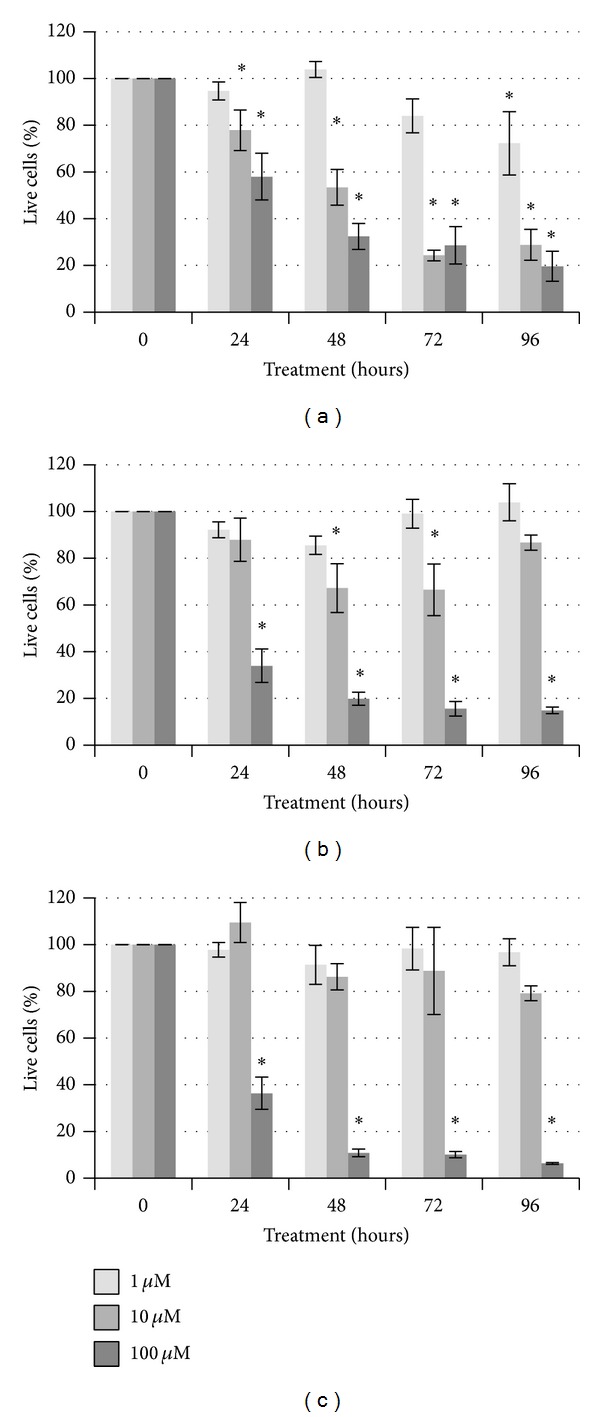
Viability of breast cancer cells after exposure to 1 *µ*M, 10 *µ*M, or 100 *µ*M cantharidin. MDA-MB-231 (a), SK-BR-3 (b), or MCF-7 (c) cells were treated for up to 96 hours with cantharidin or DMSO vehicle control. Significant differences compared to the vehicle control (*P < 0.05) are indicated.

Cantharidin has been sought out previously as a potential chemotherapy drug, as it shows a high level of toxicity in a variety of cancerous cell lines [24, 26, 27, 47, 48]. However, limited direct comparisons to current chemotherapeutics have been made. Chang et al. [49] report an IC_50_ of 50 *µ*M for cantharidin in MCF-7 cells, thus we selected a range of 1 to 100 *µ*M for our study. Unlike paclitaxel and cyclophosphamide, our data show that cantharidin was able to significantly reduce viability in all three cell lines (Figure 3). At 1 *µ*M cantharidin, up to thirty percent cell death was observed in the MDA-MB-231 cells (Figure 3(a)), and there was more than fifty percent cell death after at least 72 hours of treatment in the higher concentrations. In the other two cell lines, statistically significant cell death compared to the vehicle control was not consistently observed until the 100 *µ*M concentration. However, this is significantly more cell death than was observed for the other two toxins in each of the cell lines examined; when concentration is not factored in, there is a statistically more death for all three cell lines for cantharidin than either cyclophosphamide or paclitaxel (*P* < 0.001).

While cyclophosphamide and paclitaxel show limited toxicity at their reported IC_50_ levels, our data indicate that cantharidin can reduce viability significantly regardless of cell milieu. Thus, cantharidin holds potential as a better chemotherapeutic option for cancers with a variety of genetic backgrounds. As cantharidin is effective without being metabolized by the liver, it could be injected locally into the tumor rather than being dosed systematically. However, we must consider that even with localized dosage, there is still a systemic risk of toxicity. Conflicting reports of cantharidin toxicity to the gastrointestinal and renal systems make the choice to utilize cantharidin a difficult one. There are studies that indicate that, in both* in vitro* environment using pancreatic cells and several clinical trials, cantharidin is more toxic to cancerous cells than noncancerous [28, 50, 51]. However, historical evidence dictates that the kidney and liver have shown particular sensitivity to cantharidin, due to their physiological role in clearance [31, 32, 52]. To reduce the potential toxicity of cantharidin to noncancerous cells, our next goal is to collaborate with chemists at our institution to develop a drug delivery mechanism which will protect the normal tissue and allow for directed cell death only in the area of the tumor. Nanoparticles hold much promise as an encapsulating agent [53, 54]. Additionally, nanoparticles reduce the severity of many of the side effects of conventional chemotherapy [55, 56]. Thus, future studies will focus upon creating a nontoxic shell from liposomes and hollow gold nanoparticles that will encapsulate the cantharidin to reduce its toxicity should it come in contact with nontumor cells. Once synthesized, a release mechanism will be identified to ensure delivery of the toxin to only the tumor. Understanding the interaction of these nanoparticles with cancerous and noncancerous cells will allow improved diagnosis and treatment in cancer research, including the delivery of chemotherapy and targeting of tumors.

## 4. Conclusion

In each of our cell types examined, MDA-MB-231, SK-BR-3, and MCF-7 cells, we observed a high decrease in cell viability after cantharidin was applied when comparing it to the untreated control, reaching more than 80% cell death for each of the cell types after 96 hours of treatment with 100 *µ*M cantharidin. This is substantially more death than was observed for either paclitaxel or cyclophosphamide, two commonly prescribed chemotherapy agents. Thus, we have identified a more potent toxin that may be utilized for local, rather than systemic, dosing.

## References

[1] Group USCSW (2013). United states cancer statistics: 1999–2010 incidence and mortality web-based report.

[2] Brollo J, Curigliano G, Disalvatore D (2013). Adjuvant trastuzumab in elderly with HER-2 positive breast cancer: a systematic review of randomized controlled trials. *Cancer Treatment Reviews*.

[3] Higgins MJ, Baselga J (2011). Targeted therapies for breast cancer. *Journal of Clinical Investigation*.

[4] Lehmann BD, Bauer JA, Chen X (2011). Identification of human triple-negative breast cancer subtypes and preclinical models for selection of targeted therapies. *Journal of Clinical Investigation*.

[5] Osborne CK, Neven P, Dirix LY (2011). Gefitinib or placebo in combination with tamoxifen in patients with hormone receptor-positive metastatic breast cancer: a randomized phase II study. *Clinical Cancer Research*.

[6] O'Shaughnessy J, Osborne C, Pippen JE (2011). Iniparib plus chemotherapy in metastatic triple-negative breast cancer. *The New England Journal of Medicine*.

[7] Ribi K, Aldridge J, Phillips K-A (2012). Subjective cognitive complaints one year after ceasing adjuvant endocrine treatment for early-stage breast cancer. *The British Journal of Cancer*.

[8] Pang H, Cai L, Yang Y, Chen X, Sui G, Zhao C (2011). Knockdown of osteopontin chemosensitizes MDA-MB-231 cells to cyclophosphamide by enhancing apoptosis through activating p38 MAPK pathway. *Cancer Biotherapy and Radiopharmaceuticals*.

[9] Padmanabhan N, Howell A, Rubens RD (1986). Mechanism of action of adjuvant chemotherapy in early breast cancer. *The Lancet*.

[10] Richards MA, O'Reilly SM, Howell A (1990). Adjuvant cyclophosphamide, methotrexate, and fluorouracil in patients with axillary node-positive breast cancer: an update of the Guy's/Manchester trial. *Journal of Clinical Oncology*.

[11] Horwitz SB (1994). Taxol (paclitaxel): mechanisms of action. *Annals of Oncology*.

[12] Subramanian IV, Devineni S, Ghebre R (2011). AAV-P125A-endostatin and paclitaxel treatment increases endoreduplication in endothelial cells and inhibits metastasis of breast cancer. *Gene Therapy*.

[13] Rowinsky EK, Donehower RC (1995). Paclitaxel (taxol). *The New England Journal of Medicine*.

[14] Evans WE, McLeod HL (2003). Pharmacogenomics—drug disposition, drug targets, and side effects. *The New England Journal of Medicine*.

[15] Shapiro CL, Recht A (2001). Side effects of adjuvant treatment of breast cancer. *The New England Journal of Medicine*.

[16] Wu CH, Yang CH, Lee JN, Hsu SC, Tsai EM (2001). Weekly and monthly regimens of paclitaxel and carboplatin in the management of advanced ovarian cancer. A preliminary report on side effects. *International Journal of Gynecological Cancer*.

[17] Sitzia J, Huggins L (1998). Side effects of cyclophosphamide, methotrexate, and 5-fluorouracil (CMF) chemotherapy for breast cancer. *Cancer Practice*.

[18] Cella D, Wang M, Wagner L, Miller K (2011). Survival-adjusted health-related quality of life (HRQL) among patients with metastatic breast cancer receiving paclitaxel plus bevacizumab versus paclitaxel alone: results from eastern cooperative oncology group study 2100 (E2100). *Breast Cancer Research and Treatment*.

[19] Iwamoto T (2013). Clinical application of drug delivery systems in cancer chemotherapy: review of the efficacy and side effects of approved drugs. *Biological and Pharmaceutical Bulletin*.

[20] Andre F, Hatzis C, Anderson K (2007). Microtubule-associated protein-tau is a bifunctional predictor of endocrine sensitivity and chemotherapy resistance in estrogen receptor-positive breast cancer. *Clinical Cancer Research*.

[21] Giannakakou P, Sackett DL, Kang Y-K (1997). Paclitaxel-resistant human ovarian cancer cells have mutant *β*-tubulins that exhibit impaired paclitaxel-driven polymerization. *The Journal of Biological Chemistry*.

[22] Yu D, Liu B, Jing T (1998). Overexpression of both p185(c-erbB2) and p170(mdr-1) renders breast cancer cells highly resistant to taxol. *Oncogene*.

[23] Till JS, Majmudar BN (1981). Cantharidin poisoning. *Southern Medical Journal*.

[24] Rauh R, Kahl S, Boechzelt H, Bauer R, Kaina B, Efferth T (2007). Molecular biology of cantharidin in cancer cells. *Chinese Medicine*.

[25] Li Y-M, Casida JE (1992). Cantharidin-binding protein: identification as protein phosphatase 2A. *Proceedings of the National Academy of Sciences of the United States of America*.

[26] Kuo J-H, Chu Y-L, Yang J-S (2010). Cantharidin induces apoptosis in human bladder cancer TSGH 8301 cells through mitochondria-dependent signal pathways. *International Journal of Oncology*.

[27] Huang WW, Ko SW, Tsai HY (2011). Cantharidin induces G2/M phase arrest and apoptosis in human colorectal cancer colo 205 cells through inhibition of CDK1 activity and caspase-dependent signaling pathways. *International Journal of Oncology*.

[28] Li W, Xie L, Chen Z (2010). Cantharidin, a potent and selective PP2A inhibitor, induces an oxidative stress-independent growth inhibition of pancreatic cancer cells through G2/M cell-cycle arrest and apoptosis. *Cancer Science*.

[29] Wang G-S (1989). Medical uses of mylabris in ancient China and recent studies. *Journal of Ethnopharmacology*.

[30] Wang C-C, Wu C-H, Hsieh K-J, Yen K-Y, Yang L-L (2000). Cytotoxic effects of cantharidin on the growth of normal and carcinoma cells. *Toxicology*.

[31] Sandroni P (2001). Aphrodisiacs past and present: a historical review. *Clinical Autonomic Research*.

[32] Karras DJ, Farrell SE, Harrigan RA, Henretig FM, Gealt L (1996). Poisoning from “spanish fly” (cantharidin). *The American Journal of Emergency Medicine*.

[33] Sargent JM, Taylor CG (1989). Appraisal of the MTT assay as a rapid test of chemosensitivity in acute myeloid leukaemia. *British Journal of Cancer*.

[34] Huttunen KM, Raunio H, Rautio J (2011). Prodrugs-from serendipity to rational design. *Pharmacological Reviews*.

[35] Pratt RM, Willis WD (1985). In vitro screening assay for teratogens using growth inhibition of human embryonic cells. *Proceedings of the National Academy of Sciences of the United States of America*.

[36] Haq MR, Ashraf S, Malik CP, Ganie AA, Shandilya U (2011). *In vitro* cytotoxicity of *Parthenium hysterophorus* extracts against human cancerous cell lines. *Journal of Chemical and Pharmaceutical Research*.

[37] Cohen JL, Jao JY (1970). Enzymatic basis of cyclophosphamide activation by hepatic microsomes of the rat.. *Journal of Pharmacology and Experimental Therapeutics*.

[38] Franke HR, Kole S, Ciftci Z, Haanen C, Vermes I (2003). In vitro effects of estradiol, dydrogesterone, tamoxifen and cyclophosphamide on proliferation vs. death in human breast cancer cells. *Cancer Letters*.

[39] Henderson IC, Berry DA, Demetri GD (2003). Improved outcomes from adding sequential paclitaxel but not from escalating doxorubicin dose in an adjuvant chemotherapy regimen for patients with node-positive primary breast cancer. *Journal of Clinical Oncology*.

[40] Holmes FA, Walters RS, Theriault RL (1991). Phase II trial of taxol, an active drug in the treatment of metastatic breast cancer. *Journal of the National Cancer Institute*.

[41] Robert NJ, Diéras V, Glaspy J (2011). RIBBON-1: randomized, double-blind, placebo-controlled, phase III trial of chemotherapy with or without bevacizumab for first-line treatment of human epidermal growth factor receptor 2-negative, locally recurrent or metastatic breast cancer. *Journal of Clinical Oncology*.

[42] Merlin JL, Barberi-Heyob M, Bachmann N (2002). In vitro comparative evaluation of trastuzumab (Herceptin) combined with paclitaxel (Taxol) or docetaxel (Taxotere) in HER2-expressing human breast cancer cell lines. *Annals of Oncology*.

[43] Nakayama S, Torikoshi Y, Takahashi T (2009). Prediction of paclitaxel sensitivity by CDK1 and CDK2 activity in human breast cancer cells. *Breast Cancer Research*.

[44] Lai D, Ho KC, Hao Y, Yang X (2011). Taxol resistance in breast cancer cells is mediated by the hippo pathway component TAZ and its downstream transcriptional targets Cyr61 and CTGF. *Cancer Research*.

[45] Lovat F, Ishii H, Schiappacassi M (2014). LZTS1 downregulation confers paclitaxel resistance and is associated with worse prognosis in breast cancer. *Oncotarget*.

[46] Wang X-X, Zhu Z, Su D (2011). Down-regulation of leucine zipper putative tumor suppressor 1 is associated with poor prognosis, increased cell motility and invasion, and epithelial-to-mesenchymal transition characteristics in human breast carcinoma. *Human Pathology*.

[47] Sakoff JA, Ackland SP, Baldwin ML, Keane MA, McCluskey A (2002). Anticancer activity and protein phosphatase 1 and 2A inhibition of a new generation of cantharidin analogues. *Investigational New Drugs*.

[48] Sakoff JA, McCluskey A (2004). Protein phosphatase inhibition: structure based design. Towards new therapeutic agents. *Current Pharmaceutical Design*.

[49] Chang C-C, Liu D-Z, Lin S-Y (2008). Liposome encapsulation reduces cantharidin toxicity. *Food and Chemical Toxicology*.

[50] Deng LP, Dong J, Cai H, Wang W (2013). Cantharidin as an antitumor agent: a retrospective review. *Current Medicinal Chemistry*.

[51] Zhan Y-P, Huang X-E, Cao J (2012). Clinical study on safety and efficacy of qinin (cantharidin sodium) injection combined with chemotherapy in treating patients with gastric cancer. *Asian Pacific Journal of Cancer Prevention*.

[52] Prasad SB, Verma AK (2013). Cantharidin-mediated ultrastructural and biochemical changes in mitochondria lead to apoptosis and necrosis in murine dalton’s lymphoma. *Microscopy and Microanalysis*.

[53] Jain PK, ElSayed IH, El-Sayed MA (2007). Au nanoparticles target cancer. *Nano Today*.

[54] Unak G, Ozkaya F, Ilker Medine E (2012). Gold nanoparticle probes: design and in vitro applications in cancer cell culture. *Colloids and Surfaces B: Biointerfaces*.

[55] Alexiou C, Schmid RJ, Jurgons R (2006). Targeting cancer cells: magnetic nanoparticles as drug carriers. *European Biophysics Journal*.

[56] Han W, Wang S, Liang R (2013). Non-ionic surfactant vesicles simultaneously enhance antitumor activity and reduce the toxicity of cantharidin. *International Journal of Nanomedicine*.

